# Habitat Suitability of *Danaus genutia* Based on the Optimized MaxEnt Model

**DOI:** 10.3390/insects15120971

**Published:** 2024-12-05

**Authors:** Jun Yao, Chengli Zhou, Wenquan Wang, Yangyang Li, Ting Du, Lei Shi

**Affiliations:** 1 Key Laboratory of Breeding and Utilization of Resource Insects of National Forestry and Grassland Administration, Institute of Highland Forest Science, Chinese Academy of Forestry, Kunming 650224, China; eyaojun@hotmail.com (J.Y.); buttzhou@163.com (C.Z.); eggbasten@foxmail.com (W.W.); lyangyang304@gmail.com (Y.L.); duting_x_y@163.com (T.D.); 2 Yunnan Key Laboratory of Breeding and Utilization of Resource Insects, Kunming 650224, China; 3 Graduate School, Nanjing Forestry University, Nanjing 210037, China

**Keywords:** *Danaus genutia*, *Cynanchum annularium*, potential distribution, environmental factors, interspecific relationships, Yuanjiang River Valley

## Abstract

*Danaus genutia*, commonly known as the tiger butterfly, is a visually appealing species in the Danaidae family. As it is not currently classified as endangered, it is excluded from key protected species lists at national and local levels, limiting focus on its population and habitat status, which may result in it being overlooked in local butterfly conservation initiatives. Yunnan, characterized by high butterfly diversity, presents an ideal region for studying habitat suitability for *D. genutia*, which may support the conservation of regional biodiversity. This study employs the MaxEnt ecological niche model, predictions regarding suitable habitat distribution, and trends for *D. genutia* and identifying primary environmental factors influencing their distribution. The results indicate that the niche model that includes interspecies relationships provides a distribution prediction closely aligned with the observed range of *D. genutia*. Under current climatic conditions, highly suitable habitats for both *D. genutia* and its host plant, *Cynanchun annularium*, are located predominantly in the Yuanjiang River Valley. Optimal conditions occur at average annual temperatures of 19.80–22 °C for *D. genutia* and 22–24 °C for *C. annularium*. The distribution range of *C. annularium* is a vital biological factor limiting *D. genutia*’s habitat. By 2040, projections under four future climate scenarios indicate a potential increase in the total area of suitable habitats for *D. genutia*, with a general trend of northward expansion.

## Simple Summary

This study aims to determine the potential geographic distribution of *Danaus genutia* in Yunnan Province, identify the main environmental factors affecting its distribution, analyze habitat suitability, and predict the changes in the distribution pattern of suitable areas under current and future climate change scenarios. The results show that climate is an important factor influencing the distribution of both *D. genutia* and its host plant, *Cynanchum annularium*. The Yuanjiang River Valley in Yunnan, with its unique topography, geomorphology, and climatic conditions, provides suitable habitats for the butterfly. A strong interaction exists between *D. genutia* and *C. annularium*, and the decline of the host plant may be an important factor contributing to the decrease in butterfly populations in the Yuanjiang River Valley. By 2040, under four future climate scenarios, the total area of suitable habitat for *D. genutia* is projected to increase, with an overall trend of northward expansion. This study provides scientific theoretical support for the conservation of *D. genutia* natural resources.

## 1. Introduction

Yunnan Province, recognized as China’s richest region in biodiversity, has a unique topography and geographical location that make it a major passage for the exchange of biological species between Eurasia. In the world’s animal geographical division, it is located at the intersection of the Palearctic and Oriental regions. Its biological flora reflect the characteristics of the intersection between the north and south, complex geographical elements, and prominent endemic components. It holds the top position nationwide for biodiversity and is designated as one of the 17 key biodiversity areas in China, as well as one of the 34 global hotspots for species richness [1,2]. It has gathered a variety of climate types and biological community types equivalent to the southern subtropical, mid-subtropical, northern subtropical, warm temperate, temperate, cold temperate, and cold zones of the Northern Hemisphere. It boasts more than 20 ecosystems, making it the region with the richest ecosystem types per unit area in the world. Hosting the first phase of the 15th Conference of the Parties to the United Nations Convention on Biological Diversity (COP15), Yunnan has biodiversity conservation initiatives that are significant both nationally and internationally. Yunnan hosts an impressive 1300 butterfly species across six families and 356 genera, representing about 79.8% of the total butterfly genera and 58.6% of species found in China, highlighting its unique butterfly diversity [3]. While some butterfly diversity surveys have been conducted in China, they largely focus on species counts and classifications in specific protected areas, lacking large-scale systematic studies and comprehensive consideration of climate change impacts. Climate change has affected organisms in various global regions and is expected to continue influencing ecosystems in the coming decades [4]. The tiger butterfly, *Danaus genutia* (Cramer, 1779), classified under family Danaidae, and genus *Danaus* Kluk [5], is known for its striking wing patterns that resemble tiger stripes and its slow, elegant flight, making it aesthetically appealing, with no reported damage to agricultural or forestry plants. However, it is not currently considered endangered, nor is it listed among protected species at the national or local levels, leading to limited monitoring of its populations and habitat conditions, and consequently, it may be overlooked in local butterfly conservation strategies.

Species distribution models (SDMs) utilize occurrence and environmental data to estimate a species’ ecological niche through specific algorithms, providing insights into habitat preferences and predicting habitat suitability. These results are often interpreted as probabilities of species occurrence, with niche theory serving as the primary conceptual foundation for SDMs [6]. Depending on research objectives, SDMs are sometimes termed ecological niche models (ENMs) [7], effectively elucidating the role of environmental variables in shaping species distributions [8]. By quantifying correlations between species distribution points and various environmental variables, ENMs predict regions that align with species’ ecological needs [9]. Among ENM algorithms, the MaxEnt model is especially favored due to its rapid computation and high predictive accuracy [10]. With advances in species distribution prediction methods, climate change impacts are increasingly integrated as essential factors in model projections [11]. Accurate predictions also rely on high-quality foundational data related to biological factors [12]. Recent studies on the interactions between phytophagous insects, such as butterflies, and their host plants have begun incorporating interspecies relationships as constraints in prediction models, demonstrating improved prediction accuracy and greater ecological relevance in distribution outcomes [13].

In recent years, the MaxEnt ecological niche model has been applied in the study of rare butterfly species [14,15,16], but there has been no precedent for conducting conservation gap analysis based on quantitative simulations of *D. genutia*’s suitable habitat. Current research on *D. genutia* primarily focuses on morphological characteristics, life history, and behavior [17,18,19]. This study incorporates the interaction between *D. genutia* and its host plant, *Cynanchum annularium* (Roxb.) Liede & Khanum, within an optimized MaxEnt model to simulate suitable habitats under present and projected climatic conditions, forecasting potential distributions in Yunnan. It further identifies key environmental factors influencing *D. genutia’*s potential distribution, contributing scientific insights to support conservation of its natural habitats.

## 2. Materials and Methods

### 2.1. Collection and Processing of Distribution Data for D. genutia and Its Host Plant

**Figure 1:**
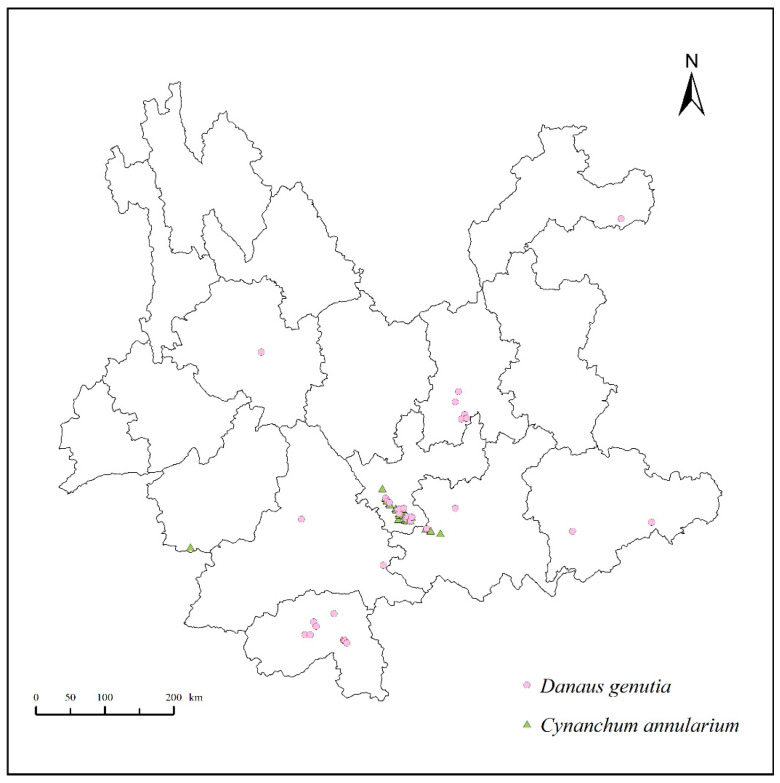
The distribution records of *Danaus genutia* and *Cynanchum annularium* in Yunnan.

Distribution data for *D. genutia* and its host plant, *C. annularium*, in Yunnan were compiled through field surveys and supplemented with records from the Global Biodiversity Information Facility (https://www.gbif.org, accessed on 3 April 2024) and iNaturalist (https://www.inaturalist.org, accessed on 3 April 2024) [10]. A total of 87 distribution points were identified for *D. genutia* (13 from field surveys and 74 from online databases), while 28 points were recorded for *C. annularium* (27 from field surveys and 1 from a database). To mitigate spatial autocorrelation among these distribution points, a spatial filtering method was applied [20]. This approach utilized ENMTools 1.4 to eliminate redundant distribution points within a 30 arc-second grid, equivalent to about 1 km × 1 km [21]. Following adjustments to address sampling bias, 37 valid records for *D. genutia* and 27 for *C. annularium* were retained for further analysis (Figure 1).

### 2.2. Model Structure Design and Establishment

The distribution of butterflies is shaped by both biotic and abiotic influences, such as vegetation, climate, topography, and human activities. For this study, accounting for the interspecies relationship between *D. genutia* and its host plant, *C. annularium*, we selected 27 environmental variables to develop four distinct model structures (Table 1) [16]:

Model M-I: This model uses the distribution data of *C. annularium* and 26 abiotic factors, including 19 bioclimatic factors, 3 topographic factors (elevation, slope, and aspect), 1 natural environmental factor (water systems), and 3 human activity factors (settlements, facilities, and roads).

Model M-II: This model is based on the distribution data of *D. genutia* and includes 26 abiotic factors.

Model M-III: This model integrates *D. genutia* distribution data with 2 biotic factors—vegetation and the potential suitable distribution of *C. annularium* (predicted from Model M-I).

Model M-IV: This comprehensive model incorporates *D. genutia* distribution data alongside 26 abiotic factors and 2 biotic factors.

### 2.3. Acquisition and Screening of Environmental Factor Data

Current and future climate data were sourced from the WorldClim database (https://www.worldclim.org, accessed on 20 May 2024). The current climate data, spanning 1970–2000 (version 2.1, released January 2020), includes 19 bioclimatic variables at a resolution of 30 arc-seconds (about 1 km × 1 km). Future climate data were obtained from four Shared Socioeconomic Pathways (SSPs) scenarios—SSP1-2.6, SSP2-4.5, SSP3-7.0, and SSP5-8.5—using the BCC_CSM2_MR model developed by the National Climate Center in Beijing. SSP1-2.6 represents a low radiative forcing scenario under a sustainable development (SSP1) pathway, stabilizing at 2.6 W/m^2^ by 2100, while SSP2-4.5 indicates a moderate radiative forcing scenario under a moderate development (SSP2) pathway, stabilizing at 4.5 W/m^2^ by 2100. SSP3-7.0 reflects a moderate to high radiative forcing scenario under a regional development (SSP3) pathway, stabilizing at 7.0 W/m^2^ by 2100, and SSP5-8.5 denotes a high radiative forcing scenario under a conventional development (SSP5) pathway, stabilizing at 8.5 W/m^2^ by 2100 [22]. To ensure spatial consistency, future climate data with a 2.5 arc-minute resolution (about 4.5 km × 4.5 km) were resampled to 30 arc-seconds (about 1 km × 1 km) using the nearest neighbor allocation method in ArcGIS 10.6, aligning it with the spatial resolution of the current climate data. Elevation data were obtained from the Resource and Environmental Science Data Platform (https://www.resdc.cn, accessed on 20 May 2024), and slope and aspect data were extracted using ArcGIS 10.6. Vegetation type, water systems, roads, settlements, and facility point data were sourced from the 1:1,000,000 public version of the Basic Geographic Information Database (2021) from the National Catalogue Service for Geographic Information (http://www.webmap.cn, accessed on 20 May 2024). ArcGIS 10.6 was used to convert the data into raster format, standardizing the boundary, coordinate system, and pixel size for all environmental variables and converting them into “asc” format. To avoid model inaccuracy due to an excessive number of environmental variables, a preliminary screening was conducted. Environmental factors with contributions to the model exceeding 1% were selected, and correlation analysis in ENMTools retained factors with a correlation coefficient |r| < 0.80 that also had significant contributions to the model [21]. This screening process identified environmental factors influencing the potential distribution of *D. genutia* and its host plant for model development (Table 2).

### 2.4. Model Optimization and Parameter Settings

**Figure 2:**
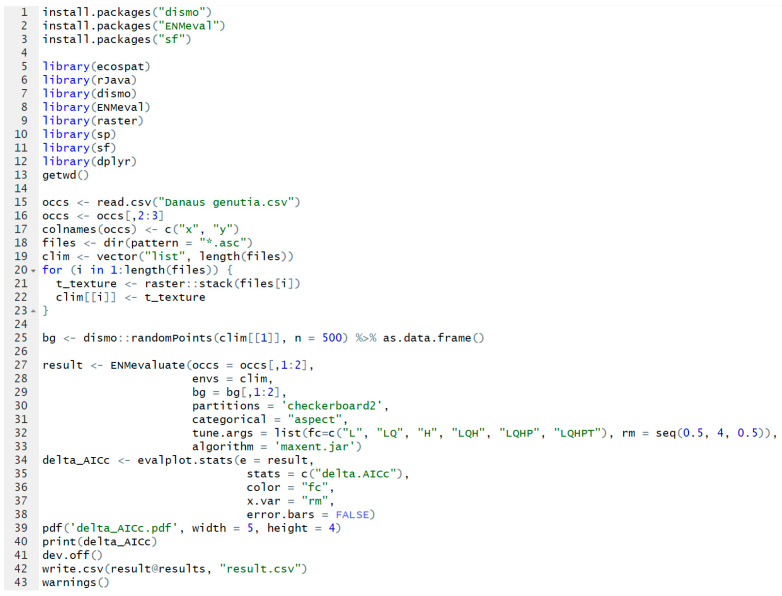
Optimization of MaxEnt model parameters using the ENMeval package in R.

To enhance the accuracy and reliability of the MaxEnt model in predicting *D. genutia* and its host plant, this study optimized the Regularization Multiplier (RM) and Feature Combination (FC) parameters using the ENMeval 2.0.4 package in R 4.4.1 [23]. Six feature combinations (FC) were established: L, LQ, H, LQH, LQHP, and LQHPT, while the RM was adjusted from 0.5 to 4.0 in 8 increments of 0.5 [24]. The corrected Akaike Information Criterion (AICc) values (delta AICc) for these combinations were calculated using the checkerboard 2 method in R, with the parameter set achieving the lowest delta AICc value selected to minimize model overfitting [25] (Figure 2).

**Figure 3:**
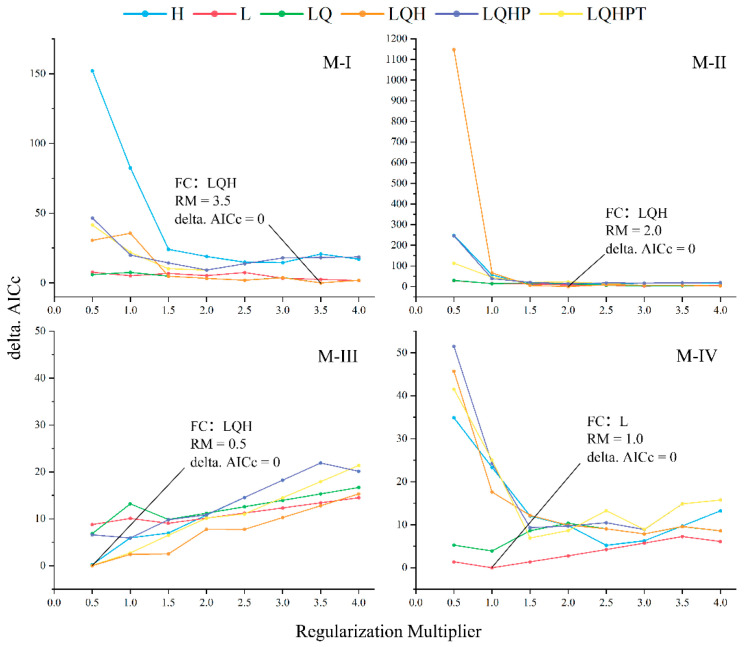
Optimization results for the model by ENMeval (H—Hinge, L—Linear, Q—Quadratic, P—Product, T—Threshold).

The optimized parameters for each model were as follows: Model M-I utilized an RM of 3.5 and FC of LQH, Model M-II had an RM of 2.0 and FC of LQH, Model M-III was optimized with an RM of 0.5 and FC of LQH, and Model M-IV employed an RM of 1.0 and FC of L (Figure 3). The random test data percentage was set at 25%, with the remaining 75% used for training. A random seed was selected, and bootstrap resampling was conducted 10 times, with a maximum of 500 iterations, outputting results in Logistic format.

### 2.5. Model Accuracy Assessment

The Receiver Operating Characteristic (ROC) curve was employed to assess the predictive accuracy of the models, with the Area Under the Curve (AUC) used as the evaluation index. According to the assessment scale, AUC values above 0.9 signify excellent performance, values from 0.8 to 0.9 indicate good performance, 0.7 to 0.8 reflect average performance, 0.6 to 0.7 denote poor performance, and values below 0.6 represent model failure [26].

### 2.6. Suitable Habitat Classification

Habitat suitability was classified based on the Logistic Threshold (LT) calculated by MaxEnt, with the lowest presence threshold serving as the criterion for differentiating habitat types [27]. Using reclassification tools in ArcGIS, habitat suitability was categorized into four levels: unsuitable habitat (0-LT), lowly suitable habitat (LT-0.4), moderately suitable habitat (0.4–0.7), and highly suitable habitat (0.7–1.0) [14]. The ArcGIS software was then used to calculate the proportion of different suitable areas from the raster layer attribute table, ultimately determining the area of each suitable habitat and creating predictive maps of the potential suitable habitats of *D. genutia* in Yunnan under current and future climate conditions.

## 3. Results

### 3.1. Model Accuracy Evaluation

**Figure 4:**
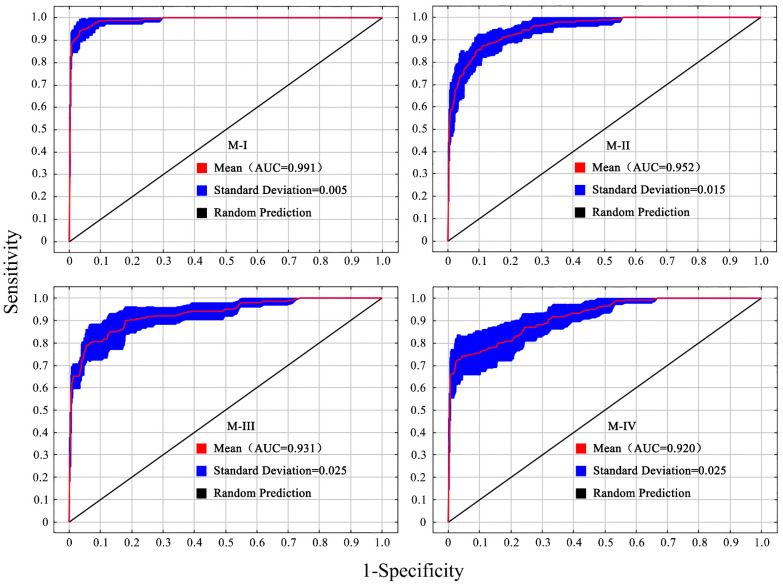
ROC evaluation curve of MaxEnt.

The accuracy assessment of the MaxEnt model following parameter optimization revealed AUC values of 0.991 ± 0.005, 0.952 ± 0.015, 0.931 ± 0.025, and 0.920 ± 0.025 for the four structural models, respectively. All values exceeded 0.9, indicating that the prediction results were accurate and demonstrated high reliability (Figure 4).

### 3.2. Potential Suitable Habitats for C. annularium and D. genutia Under Current Climate Conditions

**Figure 5:**
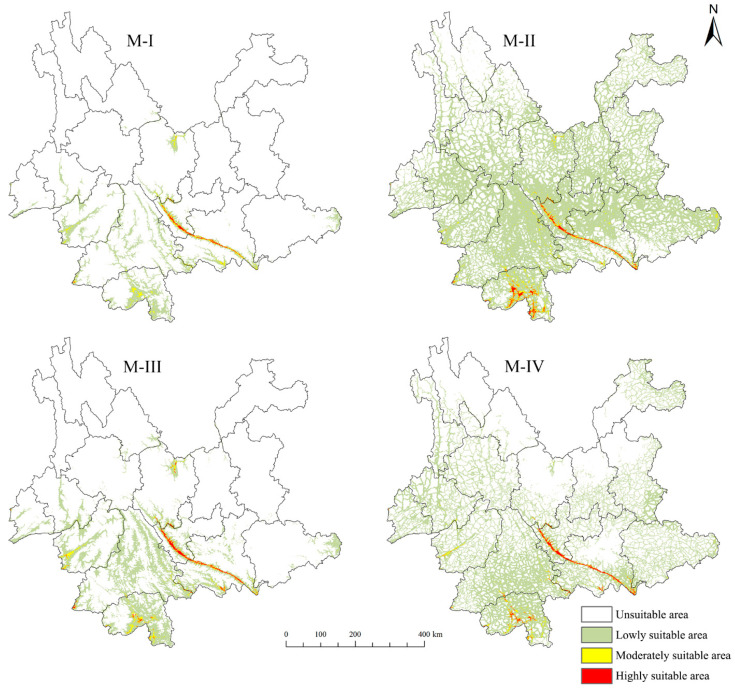
Prediction of potential suitable habitat distribution of *C. annularium* and *D. genutia* with different model structures under current climate conditions in Yunnan.

As presented in Table 3 and Figure 5, the suitable habitat area predicted by Model M-II is larger than that of Model M-I, indicating that when solely abiotic factors are considered, the potential distribution area for *D. genutia* exceeds that of its host plant, *C. annularium*. Additionally, the total suitable area for Model M-IV lies between those of Models M-II and M-III, suggesting that predictions from Model M-IV, which incorporates both biotic and abiotic factors, provide a more credible assessment of habitat suitability.

Models M-I and M-II reveal that, when considering only abiotic factors, the highly suitable habitats for both *C. annularium* and *D. genutia* are located in the Yuanjiang River Valley, suggesting that these species share similar climatic and topographic requirements. Model M-I predictions show that the suitable habitat for *C. annularium* is primarily concentrated in southwestern Yunnan, particularly around Jinghong City and Mengla County in Xishuangbanna Prefecture, with sparse distributions in other regions. Additional suitable areas are located in Yuanmou County of Chuxiong Prefecture, Funing County of Wenshan Prefecture, and scattered areas in Huaping and Yongsheng counties of Lijiang City. The highly suitable zone stretches along the Yuanjiang River Valley from Xinping County in Yuxi City to Hekou County in Honghe Prefecture, gradually transitioning into medium and low-suitability zones on both sides of the valley. The total suitable habitat area for *C. annularium* in Yunnan Province is estimated at 2.601 × 10^4^ km^2^. In contrast, Model M-IV, which integrates both biotic and abiotic factors for *D. genutia*, predicts that most regions across the province are suitable for *D. genutia*, excluding certain areas in Diqing Prefecture, Lijiang City, northern Chuxiong Prefecture, northern Kunming City, northern Qujing City, southwestern Zhaotong City, and central Honghe Prefecture. Beyond the Yuanjiang River Valley, highly suitable habitats are found in Jinghong City and Mengla County of Xishuangbanna Prefecture, and along the borders of Jinping and Luchun counties in Honghe Prefecture, as well as Jiangcheng County in Pu’er City. Overall, *D. genutia* exhibits a broad yet fragmented suitable range across Yunnan, totaling 10.494 × 10^4^ km^2^.

### 3.3. Main Environmental Factors Influencing the Distribution of D. genutia and Host Plant

In Model M-I, the cumulative contributions of four climatic factors were as follows: annual average temperature (Bio1) contributed 85.8%, isothermality (Bio3) contributed 4.5%, annual precipitation (Bio12) contributed 2.6%, and daily temperature range (Bio2) contributed 0.6%, totaling 93.5%. The single-factor replacement importance of Bio1 alone reached 94.1%. Topographic factor aspect and natural factor hydrology contributed 3.3% and 3.1%, respectively. In Model M-IV, the contribution of the potential distribution of *C. annularium* (from M-II) was markedly high at 81.3%, significantly surpassing that of other abiotic factors. Among human activity factors, roads (LRDL) showed the highest single-factor replacement importance at 51.6%. These findings indicate that annual average temperature and the presence of the host plant substantially influence the predictive outcomes of Models M-I and M-IV, while annual average temperature and road proximity emerge as the most critical factors impacting the accuracy of these models (Table 4).

**Figure 6:**
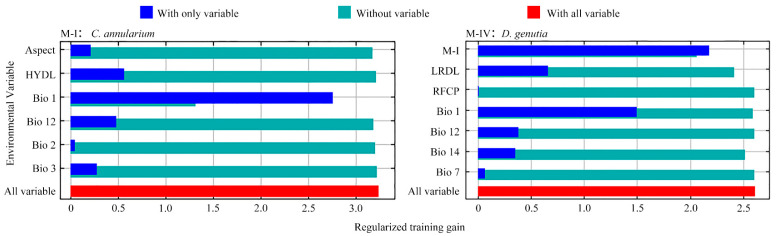
Evaluation of the importance of different environmental factors based on the Jackknife test.

The Jackknife test results show that blue bars indicate the relative importance of each environmental factor to species distribution, with longer bars signifying higher importance for the potential distribution. Green bars represent the gain value of the model contributed by other environmental factors when a specific factor is excluded. In Figure 6, Model M-I demonstrates that Bio1 (annual average temperature), HYDL (hydrology), and Bio12 (annual precipitation) are key factors impacting the potential distribution of *C. annularium*, with the gain value decreasing most rapidly upon the removal of Bio1. For Model M-IV, factors M-I, Bio1, and LRDL (road proximity) exhibit the greatest impact on the potential distribution of *D. genutia*. When M-I is excluded, the remaining six environmental factors yield the smallest gain value in Model M-IV’s prediction for *D. genutia*. Combined analysis of both models reveals that the potential distributions of *C. annularium* and *D. genutia* are both significantly influenced by two climatic factors: annual average temperature and annual precipitation. Additionally, *C. annularium* emerges as a crucial limiting factor for the potential distribution of *D. genutia*, indicating a strong interspecies interaction between the two.

**Figure 7:**
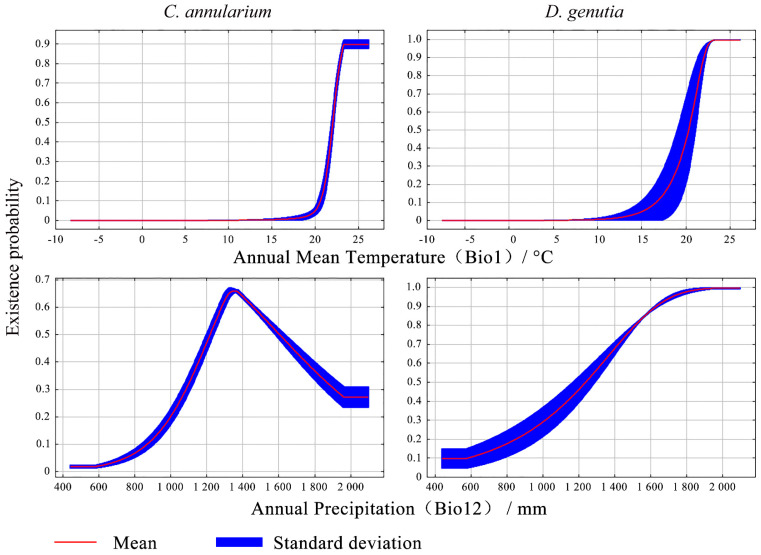
Response curves of existence probability for *C. annularium* and *D. genutia* to dominant climatic factors.

In this study, the species survival probability in the moderately suitable area exceeds 0.4, indicating that the corresponding environmental factor values support growth and development. Univariate response curves show that for *C. annularium*, optimal growth occurs at annual average temperatures between 22–24 °C and annual precipitation of 1155–1749 mm, with the highest survival probability observed at 1349 mm of annual precipitation. For *D. genutia*, suitable conditions include annual average temperatures of 19.80–22 °C and annual precipitation of 1135–1961 mm. These findings suggest that the climatic conditions necessary for the survival of both species are closely aligned (Figure 7).

### 3.4. Changes in Potential Suitable Habitats for D. genutia in Yunnan Province Under Future Climate Conditions

**Figure 8:**
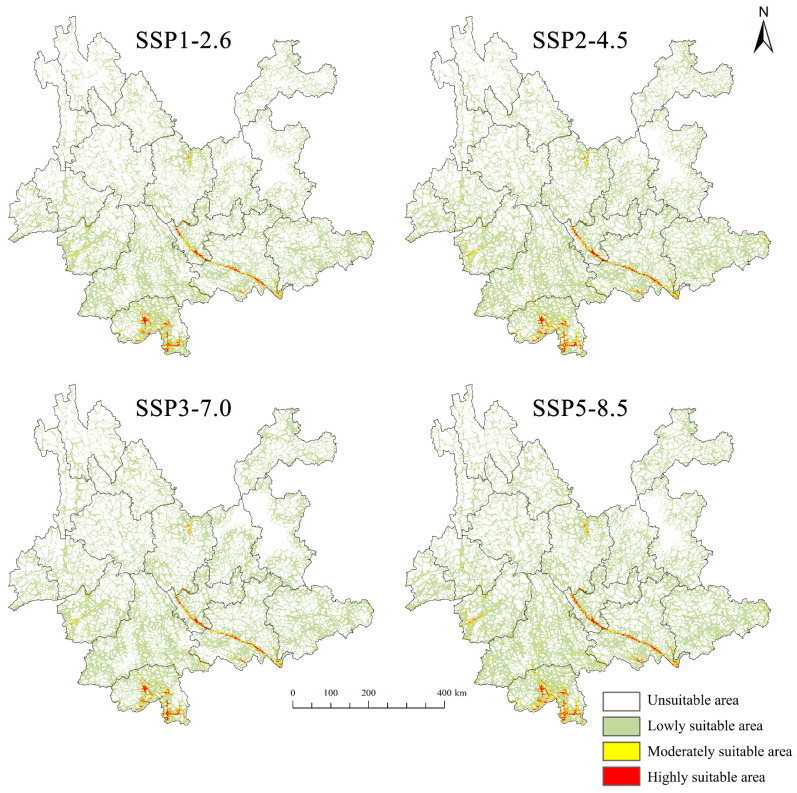
Habitat suitability for *D. genutia* under future climate scenarios.

The potential distribution of suitable habitats for *D. genutia* in Yunnan Province is influenced by climate change, with varying impacts across different climate scenarios. By 2040, under the four projected climate scenarios, the total suitable habitat area for *D. genutia* is expected to expand relative to current conditions, with synchronous increases in the three suitability levels under SSP2-4.5, SSP3-7.0, and SSP5-8.5. The SSP5-8.5 scenario, representing a more extreme climate future, predicts the fastest growth in suitable areas, adding 4.797 × 10^4^ km^2^ and bringing the total suitable habitat to 15.291 × 10^4^ km^2^, covering 38.8% of Yunnan Province. In contrast, the SSP1-2.6 scenario projects the smallest increase, with an additional 1.411 × 10^4^ km^2^, while the high suitability area shows a slight decrease of 0.022 × 10^4^ km^2^ (Table 5). Across all four climate scenarios, medium and high suitability areas remain consistent with current distributions, primarily located in the Yuanjiang River Valley, Jinghong City, and Mengla County in Xishuangbanna Prefecture (Figure 8).

**Figure 9:**
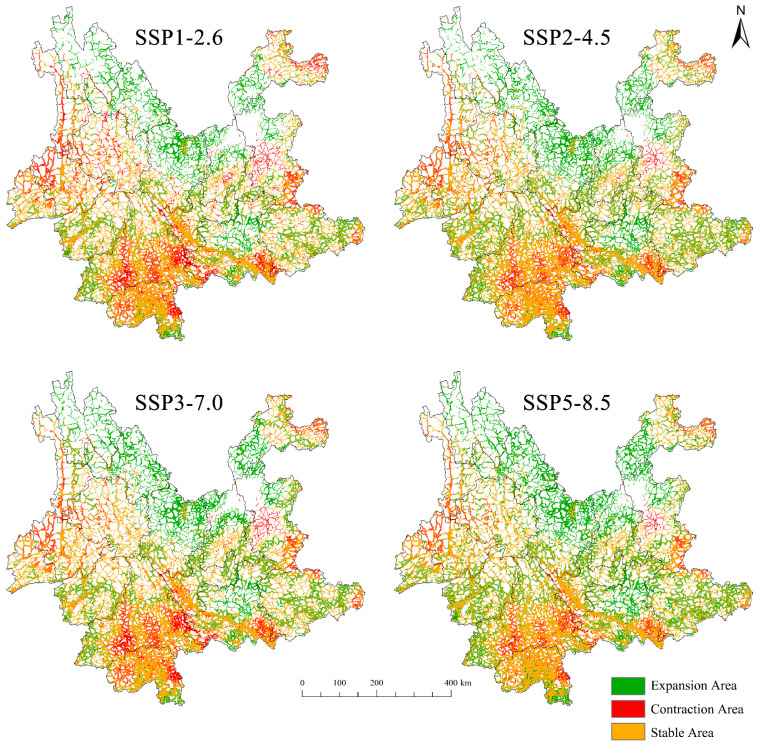
Habitat suitability for *D. genutia* under future climate change scenarios.

Under the four potential development pathways for future society, anticipated shifts in energy structure are projected to drive changes in greenhouse gas emissions, atmospheric composition, and land use, leading to a general trend of northward expansion of *D. genutia*’s suitable habitats in Yunnan Province. The primary expansion areas are located in Diqing Prefecture, Lijiang City, Chuxiong Prefecture, Kunming City, and Zhaotong City, while contraction areas are concentrated mainly in Pu’er City. Most regions in the Yuanjiang River Valley, as well as Jinghong City and Mengla County, are expected to maintain relatively stable habitat conditions (Figure 9).

## 4. Discussion

In 1948, Shannon introduced “entropy” in information theory to describe uncertainty in information, which he termed “information entropy” [28]. Building on this, Jaynes proposed the Maximum Entropy theory in 1957, suggesting from an ecological perspective that a species will occupy as much habitat as possible in the absence of limiting factors, approaching a uniform distribution [29]. In 2004, Phillips and colleagues developed the MaxEnt model based on this theory [30], now regarded as one of the most effective and widely used ecological niche models [31]. Recently, MaxEnt ecological niche models have been widely applied in biodiversity conservation fields, including rare species protection, forest pest management, and assessing climate change impacts on species distribution [32,33,34]. Niche models based on species presence data generally indicate potential species distribution [35]. Model accuracy is influenced by niche structure, parameter settings, data validity for species distribution, and environmental variable representativeness [36]. The MaxEnt model utilizes advanced machine learning algorithms, is sensitive to sampling bias, and may overfit, with robust transferability primarily under low threshold conditions. The regularization multiplier (RM) and feature combination (FC) are the main factors affecting MaxEnt model complexity and predictive outcomes [37]. Initially, the MaxEnt model’s default parameters were not tailored to insect taxa or Asian regions, focusing instead on simulating actual species distributions [38]. When applied to predict potential distributions, default MaxEnt settings can result in overfitting, lowering transferability and making predictions less reliable or interpretable [24]. The Akaike Information Criterion (AIC) evaluates model fit, with the parameter combination that minimizes the delta AICc value helping to mitigate overfitting issues [25]. This study used the ENMeval package in R to test corrected AIC values (delta AICc) for MaxEnt under varied parameter conditions, achieving high simulation accuracy, as all models displayed AUC values above 0.9.

When strong interspecies interactions exist, excluding these interactions from model construction can result in the fundamental niche of a species appearing larger than its realized niche [6]. Additionally, interspecies dynamics impact the relationship between species and climate, a key factor for accurately predicting future potential distributions [39]. This study incorporated the interspecies relationship between *D. genutia* and its host plant, *C. annularium*, establishing four predictive models with differing structures for a comparative analysis. Results indicated that considering only abiotic factors (model M-II) produces a broader potential distribution range for *D. genutia*, encompassing that of *C. annularium* (model M-I), implying that without host plant constraints, *D. genutia* could occupy a wider habitat. Additionally, significant overlap was found in medium and highly suitable areas for both species, suggesting that they share similar climatic and topographic preferences. The potential distribution range of *D. genutia* in model M-II, which excludes biotic factors, exceeds that in model M-IV, which includes both biotic and abiotic factors, corroborating previous studies. Furthermore, the distribution range in model M-IV is broader than in model M-III, which considers only biotic factors, suggesting some dispersal capability for *D. genutia*. This observation aligns with the migratory behavior of its relative, the monarch butterfly (*D. plexippus*), which undertakes long-distance migrations influenced by climate [40]. Therefore, model M-IV, which integrates interspecies relationships, offers a more realistic prediction of *D. genutia’*s potential distribution.

Climate is a crucial environmental factor influencing species distribution and growth, with water-heat conditions playing a particularly dominant role. The Jackknife test reveals that temperature and precipitation impact the potential distributions of *D. genutia* and *C. annularium*, with temperature exerting a more significant effect than precipitation. Single-factor response curves show that annual temperature ranges of 19.80–22 °C and 22–24 °C are optimal for the development of *D. genutia* and *C. annularium*, respectively, highlighting their similar temperature requirements for survival. Research demonstrates that temperature strongly affects the growth and survival of *D. genutia*, with 17.5 °C and 30.0 °C being unfavorable for larval development, while an optimal range of 20.0 °C to 27.5 °C supports larval growth [18,19]. *D. genutia* typically inhabits subtropical and tropical regions of South Asia, preferring open, sunny forest edges, hillsides, river valleys, and abandoned farmland, and overwinters as adults, indicating its adaptability to temperature variations [41]. The distribution of its host plant, *C. annularium*, is a limiting factor for *D. genutia*, influenced not only by climate but also by water systems and slope orientation. Surveys and literature confirm that *C. annularium* commonly grows in hilly scrublands, valleys, forest edges, riverside thickets, and moist forested areas [42]. According to MaxEnt model predictions, under current climatic conditions, the Yuanjiang River Valley offers highly suitable habitats for both species. The valley, lying within the southwest monsoon zone and flanked by the Ailao and Wuliang Mountains, experiences a dry, hot valley climate shaped by local atmospheric circulation and downslope winds, with an annual temperature of 23.8 °C and precipitation between 600–800 mm in areas below 1,000 m elevation [43,44]. This unique climate and topography foster suitable habitats for both *D. genutia* and *C. annularium*. Xishuangbanna Prefecture, in addition to the Yuanjiang River Valley, is a highly suitable habitat for *D. genutia*. Unlike the Yuanjiang River Valley, this region is protected by the Hengduan Mountains to the north and experiences monsoon influences from both the Indian and Pacific Oceans, creating favorable conditions including ample sunlight, limited cold winds, and high rainfall. These climatic factors result in high temperatures, substantial humidity, and minimal wind, establishing it as the region in China with the most distinct tropical characteristics, largest tropical range, and greatest concentration of tropical biodiversity. Xishuangbanna holds significant international importance as a key area for biodiversity distribution [45]. Temperature, annual precipitation, and elevation play critical roles in sustaining butterfly diversity [46]. As the lowest latitude and elevation region in Yunnan, Xishuangbanna’s hot, humid climate, intact natural vegetation, and rich water systems create an optimal environment for butterfly diversity. Notably, 382 species and subspecies have been recorded, with *D. genutia* predominantly observed in Jinghong City and Mengla County [45]. While this study’s predictions align with these observations, Xishuangbanna is only moderately suitable for *C. annularium*, and *D. genutia* shows weaker habitat suitability here than in the Yuanjiang River Valley. Although butterflies typically exhibit distinct vertical distribution patterns [47,48], this study found that elevation does not directly influence the distributions of *D. genutia* and *C. annularium*. Instead, elevation acts as a proxy for variations in temperature and precipitation, indirectly affecting the distribution of the host plant and habitat preferences of *D. genutia*, aligning with existing scholarly perspectives [49,50].

Climate change significantly impacts insect populations and distributions, and it is anticipated to increasingly drive shifts in biodiversity and species geographic ranges in the coming decades. Species generally inhabit areas with optimal climate conditions; thus, regional temperature changes are likely to alter species distributions accordingly [51]. While projected changes in suitable habitats for *D. genutia* under four future climate scenarios display variability, this study predicts an overall increase in suitable habitats, although habitat fragmentation remains a concern. The Yuanjiang River Valley and Xishuangbanna’s Jinghong and Mengla continue to be high or moderately suitable regions for *D. genutia*. These areas reflect broader climate trends, experiencing rising temperatures annually, particularly in the Yuanjiang River Valley, where the warming rate surpasses the global average [52,53,54]. Within the tolerable temperature range, species distributions can extend beyond current limits [55], suggesting that *D. genutia* may adapt to future climate variations, potentially establishing new suitable habitats. Overall, projections indicate a northward expansion of suitable habitats for *D. genutia*, aligning with global studies on butterflies, which show similar shifts in response to climate change in regions such as the UK, Finland, and North America [56,57,58]. Mechanistic studies on the northward movement of butterflies have consistently linked these shifts to rising temperatures [59,60,61]. Research by Chen et al. [17] further reveals color polymorphism among *D. genutia* larvae in the Yuanjiang population, likely influenced by gene flow from migrating individuals. The extended adult lifespan of *D. genutia* facilitates long-distance migration, akin to its relative, *D. plexippus*. Yuanjiang’s climate also supports large overwintering clusters of *D. genutia*, with populations potentially extending from eastern India to overwinter in Yunnan.

While this study identifies the Yuanjiang River Valley as a suitable habitat for both *D. genutia* and *C. annularium* under current and projected future climate conditions, recent surveys over the past decade show a marked decline in *D. genutia* sightings across many locations within the valley, indicating a decrease in wild population density. Concurrently, *C. annularium*, locally known as “sheep milk vegetable” for its milky latex and medicinal value [42,62], has suffered habitat loss due to extensive human harvesting. This study’s Jackknife test confirms that *C. annularium* significantly influences the potential distribution of *D. genutia*, suggesting a strong ecological interaction. The depletion of this host plant may thus be a critical factor in the observed decline of *D. genutia* populations in Yuanjiang River Valley. In another study on host plant selection, it was observed that *D. genutia* could also reproduce on *C. corymbosum*, a species with traditional medicinal use in Yao ethnic medicine [63]; however, due to insufficient distribution data, *C. corymbosum* was not included in this study’s analysis. By integrating host plant presence as a biological variable, this study accurately reflects *D. genutia*’s specific “host-habitat” needs, offering a scientific foundation for its conservation to mitigate further habitat degradation. Therefore, we recommend strengthening population monitoring and habitat protection management for *D. genutia*, reducing anthropogenic damage to its host plants, guiding local communities in sustainable use of natural resources, and supplementing efforts with artificial planting to restore host plants’ populations. These measures will help provide a suitable habitat for *D. genutia*, aiming to reduce the risk of local or regional extinction.

## 5. Conclusions

Climate plays a crucial role in determining the distribution of *D. genutia* and *C. annularium*, with the Yuanjiang River Valley in Yunnan Province offering an ideal habitat due to its distinctive terrain and climate. The strong ecological interaction between *D. genutia* and *C. annularium* underscores the dependency of *D. genutia* on this host plant, suggesting that the reduction of *C. annularium* may significantly contribute to the observed decline of *D. genutia* populations in the Yuanjiang River Valley.

## Figures and Tables

**Table 1:** Establishment of models for four different structures.

Model	Species	Biotic Factor	Abiotic Factor
M-I	*Cynanchum annularium*	/	Climate, Terrain, Natural environment, Human activities
M-II	*Danaus genutia*	/	Climate, Terrain, Natural environment, Human activities
M-III	*Danaus genutia*	Vegetation, M-I	/
M-IV	*Danaus genutia*	Vegetation, M-I	Climate, Terrain, Natural environment, Human activities

**Table 2:** Screening of environmental factors affecting the distribution of host plant and *D. genutia*.

Code	Environmental Factor	M-I (6)	M-I (9)	M-III (2)	M-IV (7)
Bio 1	Annual Mean Temperature	√	√	×	√
Bio 2	Mean Diurnal Range	√	√	×	×
Bio 3	Isothermality	√	×	×	×
Bio 4	Temperature Seasonality	×	√	×	×
Bio 5	Max Temperature of Warmest Month	×	×	×	×
Bio 6	Min Temperature of Coldest Month	×	×	×	×
Bio 7	Temperature Annual Range	×	√	×	√
Bio 8	Mean Temperature of Wettest Quarter	×	×	×	×
Bio 9	Mean Temperature of Driest Quarter	×	×	×	×
Bio 10	Mean Temperature of Warmest Quarter	×	×	×	×
Bio 11	Mean Temperature of Coldest Quarter	×	×	×	×
Bio 12	Annual Precipitation	√	×	×	√
Bio 13	Precipitation of Wettest Month	×	×	×	×
Bio 14	Precipitation of Driest Month	×	×	×	√
Bio 15	Precipitation Seasonality	×	×	×	×
Bio 16	Precipitation of Wettest Quarter	×	×	×	×
Bio 17	Precipitation of Driest Quarter	×	×	×	×
Bio 18	Precipitation of Warmest Quarter	×	√	×	×
Bio 19	Precipitation of Coldest Quarter	×	×	×	×
Altitude	Altitude	×	×	×	×
Slope	Slope	×	×	×	×
Aspect	Aspect	√	√	×	×
HYDL	Water system	√	×	×	×
RESP	Residential area	×	√	×	×
RFCP	Establishment points	×	√	×	√
LRDL	Road	×	√	×	√
VEGA	Vegetation type	×	×	√	×
M-I	Potential distribution of host plant	×	×	√	√

√ indicates retention; × indicates screening out; the numbers in brackets indicate the number of retained factors.

**Table 3:** Suitable habitat areas of four structure models under current climatic conditions (units: 10^4^ km^2^).

Suitable Level	M-I		M-II		M-III		M-IV	
	Area	Proportion (%)	Area	Proportion (%)	Area	Proportion (%)	Area	Proportion (%)
Highly suitable area	0.061	2.35	0.167	0.86	0.143	2.77	0.160	1.52
Moderately suitable area	0.201	7.73	0.465	2.39	0.266	5.15	0.223	2.13
Lowly suitable area	2.339	89.92	18.841	96.75	4.752	92.08	10.111	96.35
Total suitable area	2.601	100.00	19.473	100.00	5.161	100.00	10.494	100.00

**Table 4:** Percent contribution and permutation importance of environmental factors affecting the distribution of *C. annularium* and *D. genutia*.

Model	Species	Environmental Factor	Percent Contribution (%)	Permutation Importance (%)
M-I	*C. annularium*	Bio 1	85.8	94.1
		Bio 3	4.5	0.6
		Aspect	3.3	1.7
		HYDL	3.1	1.7
		Bio 12	2.6	1.4
		Bio 2	0.6	0.5
M-IV	*D. genutia*	M-II	81.3	26.7
		LRDL	9.7	51.6
		Bio 14	5.1	15.1
		Bio 12	2.2	0
		Bio 1	1.4	4.8
		RFCP	0.2	0.1
		Bio 7	0.2	1.6

**Table 5:** Area of suitable habitats of *D. genutia* at different levels under climate change (units: 10^4^ km^2^).

Suitable Level	Current	2040s			
		SSP1-2.6	SSP2-4.5	SSP3-7.0	SSP5-8.5
Highly suitable area	0.160	0.138	0.183	0.163	0.202
Moderately suitable area	0.223	0.292	0.348	0.297	0.426
Lowly suitable area	10.111	11.475	13.315	12.803	14.663
Total suitable area	10.494	11.905	13.846	13.263	15.291

## Data Availability

Data are available on request from the authors.
